# A Destroyer of Tissues: A Case of Chronic Low-Dose Radiation Exposure and the Need for Frequent Screening in Exposed Populations

**DOI:** 10.7759/cureus.72845

**Published:** 2024-11-01

**Authors:** Jordyn Mullins, Brian R Beyer, Cheri Blacksten

**Affiliations:** 1 Family Medicine, Burrell College of Osteopathic Medicine, Las Cruces, USA

**Keywords:** geiger counter, inflammation, nuclear radiation, prostatitis, soft tissue tumor

## Abstract

The long-term effects of low-dose radiation exposure are a topic that has been lightly touched on in medical literature. We present a case of a 66-year-old male, who grew up near uranium mines, with several pathologies that are believed to be well explained by his chronic exposure to low-dose radiation. We postulate that screening for etiologies with known associations with radiation may help avoid long-term complications in at-risk populations.

## Introduction

Following the bombings of Nagasaki and Hiroshima in 1945, Tomonaga began studying and documenting the incidence of leukemia in Hiroshima in the populations impacted by prolonged radiation exposure [1]. Additionally, following the events at Chernobyl in 1986, the incidence of thyroid and hematological cancers increased [2]. Notably, prolonged radiation exposure has the potential to lead to aberrant cellular activity, resulting in neoplastic processes, even amongst healthcare professionals [3,4]. Here, we present the case of a 66-year-old man who grew up in a uranium mining town that presents with several neoplasms and inflammatory changes of solid organs that have required several operations. Off-site exposure has been reported in areas where radionucleotide decay is in higher concentration due to mining, resulting in adverse health effects as a result of inhalation, ingestion, or direct contact [5]. These stochastic effects are often seen in this setting where the person may be exposed to unknown amounts of radiation, which, in the long term, may lead to cancerous development and/or other organ dysfunction. There are also deterministic impacts of radiation where there is a clear threshold amount of radiation that drives acute effects on tissue such as dermatitis, hair loss, radiation sickness, cataracts, and other acute damage to cells in several organ systems [6].

## Case presentation

A 66-year-old male presented to our primary care clinic with recent acute adrenal insufficiency that resolved with treatment with two weeks of step-down corticosteroid treatment, prostatitis for two months, chronic lower back pain, three months status post resection of a left paraspinal mass, a biopsy that confirmed actinic keratosis of the face being treated with 5-fluorouracil, and oral thrush for one week. Per an outside pathologist report, the paraspinal mass displayed areas of spindled cells with myxoid stroma that stained negative for MUC4, CD34, STAT6, and nuclear beta-catenin expression. This led to the conclusion that the mass was likely not carcinomatous nor sarcomatous; however, intramuscular myxoma or other low-grade soft tissue neoplasms could not be excluded versus reactive stroma. He had recently been discharged from the hospital following an episode of systemic inflammatory response syndrome (SIRS), resulting in acute adrenal insufficiency, for which he was treated with corticosteroids. The episode was attributed to chronic bacterial prostatitis, with positive cultures of Staphylococcus epidermidis. He was subsequently treated with 200 mg cefpodoxime given orally every 12 hours for 38 days, resulting in resolution of SIRS, and hence was discharged.

**Figure 1:**
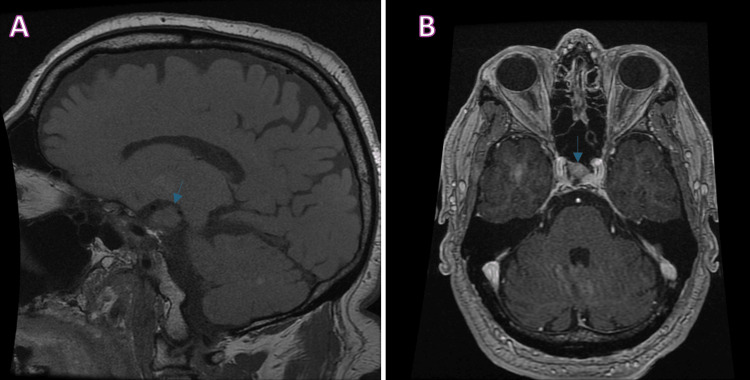
MRI brain showing pituitary macroadenoma A) T1 pre-contrast sagittal scan showing an ill-marginated lesion (blue arrow) of the sella measuring 0.8 x 1.3 cm; B) T1 post-contrast axial scan showing an ill-marginated hypoenhancing lesion (blue arrow) along the right side of the sella measuring 0.8 x 0.8 cm

At the current visit, the patient denied fever, chills, nausea, vomiting, and headaches to confirm resolution of SIRS and potential malignancies given the patient's past medical history. He noted odynophagia, nocturia, and dysuria. His past medical history was significant for a non-functional 0.8 x 0.8 x 1.3 cm pituitary macroadenoma at the age of 63 (Figure 1), prostate cancer, benign prostatic hyperplasia, migraines, hypertension, autoimmune hypothyroidism being treated with levothyroxine, degenerative disc disease of the lumbar spine with cauda equina compression, and pulmonary embolism. Chronic conditions, including, but not limited to systemic lupus erythematosus, rheumatoid arthritis, systemic sclerosis, mixed connective tissue disease, Sjogren syndrome, and other autoimmune conditions, were investigated but did not return positive results following antibody testing, including anti-thyroid peroxidase, anti-thyroglobulin, anti-cyclic citrullinated peptide, anti-rheumatoid factor, anti-Sjogren-syndrome-related antigen A and B, anti-double-stranded DNA, and anti-Smith antibodies. Multiple endocrine neoplasia’s (MEN) 1, 2A, and 2B and Li Fraumeni were also investigated but yielded negative results following genetic testing for the MEN1 gene, RET gene, and p53 gene mutations. This led to the conclusion that genetic syndromes and autoimmune disorders were likely not responsible for the current pathologies. Surgical and medical management were discussed with the patient to address the pituitary macroadenoma; however, due to its non-functional status and the lack of mass effect, both were declined. His past surgical history was significant for total thyroidectomy with parathyroidectomy; a biopsy of the prostate due to concern for cancer as a result of nocturia; painful ejaculation; elevated prostate-specific antigen at 5.6 and 5.1 ng/mL on two occasions (normal 60-69 years old: <4.5 ng/mL); right and left knee arthroscopy on two separate occasions at 59 and 61, respectively, due to meniscal tears and severe osteoarthritis; right shoulder arthroscopy; and left proximal hamstring repair of a ruptured tendon. The parathyroidectomy resulted in the removal of 3.5 glands with the reimplantation of the remaining gland in the patient’s left forearm. His current medications include lisinopril, rosuvastatin, and levothyroxine. The patient’s social history was significant for spending his childhood through his current residence in an area where radiation exposure was high due to chemical laboratories associated with nuclear mining and testing, where the patient’s father was an employee. The exposure was mostly likely through direct contact and airborne contact, based on how the patient describes his childhood. His family history is unremarkable for any genetic or autoimmune syndromes that could be linked to his current presentation with his father only having chronic obstructive pulmonary disease and died naturally at the age of 75. His mother reportedly had polycythemia vera, osteoporosis, and mild dementia and passed away at the age of 84 from natural causes. His sister has a history only significant for depression.

Laboratory values for pituitary hormones revealed prolactin of 5.9 ng/mL (normal: 2.1-17.7 ng/mL), thyroid-stimulating hormone (TSH) of <0.005 uIU/mL (normal: 0.27-4.20 uIU/mL), adrenocorticotropic hormone (ACTH) of 27 pg/mL (normal: 7-63 pg/mL), luteinizing hormone (LH) of 4.1 mIU/mL (normal: 1.7-8.6 mIU/mL), follicle-stimulating hormone (FSH) of 8.9 mIU/mL (normal: 1.5-12.4 mIU/mL), insulin-like growth factor-1 (IGF-1) of 103 ng/mL (normal: 51-187 ng/mL) (Table 1). He is currently being treated with testosterone-replacement therapy and levothyroxine due to thyroidectomy. His current chronic bacterial prostatitis confirmed by semen culture growing Escherichia coli is being treated with fosfomycin, after failing ceftriaxone for four weeks. 

**Table 1:** Pituitary hormone panel Pituitary hormone panel results to assess pituitary activity in the presence of a pituitary macroadenoma. Abbreviations: prolactin (PRL), thyroid-stimulating hormone (TSH), adrenocorticotropic hormone (ACTH), luteinizing hormone (LH), follicle-stimulating hormone (FSH), insulin-like growth factor-1 (IGF-1)

Hormone	Measured Value	Normal Value
PRL	5.9 ng/mL	2.1-17.7 ng/mL
TSH	<0.005 uIU/mL	0.27-4.20 uIU/mL
ACTH	27 pg/mL	7-63 pg/mL
LH	4.1 mIU/mL	1.7-8.6 mIU/mL
FSH	8.9 mIU/mL	1.5-12.4 mIU/mL
IGF-1	103 ng/mL	51-187 ng/mL

For this patient, the next steps in his management will be to continue to monitor his current conditions, as well as remain vigilant about other potential pathologies to arise. We are currently seeing him every six months to reassess pituitary and thyroid functions, as well as fielding any other issues that may arise. He has vocalized that he does not wish to have any more surgical procedures. We will continue to work with his specialists to manage pathologies that may otherwise usually require surgery.

## Discussion

Historically, nuclear disasters have exposed the risk and association of neoplastic development and inflammatory changes in solid organs when chronically exposed to radiation [1,2]. Awareness of low-dose radiation (<50-100 mGy) exposure during childhood, such as cancer survivors treated with radiation therapy, is an area of literature that has grown in the last decade [7]. However, a discussion of adult populations who were raised in areas where there is long-term exposure to low-dose radiation due to mining practices is warranted. In these populations, the development and progression of several pathologies, including thyroiditis, thyroid cancer, prostate cancer, breast cancer, brain tumors, gynecologic malignancies, leukemias, and lymphomas, are reported [7].

The biological effects of radiation are important in categorizing the likely exposure and impact of that exposure, as well as how these types of exposures can be avoided in the general population. Generally, there are two types of effects: deterministic and stochastic effects (Table 2) [6,8]. Deterministic effects, as mentioned earlier, rely on an acute threshold amount that immediately causes cellular detriment including erythema, hair loss, and radiation sickness. Meanwhile, stochastic effects do not have a measurable threshold and commonly lead to neoplastic development [6]. Deterministic effects are commonly observed in situations where large amounts of radiation exposure are encountered such as nuclear accidents, transportation accidents, or exposure in a scientific laboratory [8]. These exposures are much more likely to drive deterministic effects where a clear threshold will be violated, leading to acute changes in cellular makeup, which could lead to acute radiation syndrome [8]. In these instances, it is important to decontaminate, evacuate, and transport to the nearest hospital where in-hospital protocols are in place to appropriately manage and treat these patients. Conversely, stochastic effects are more commonly lower doses of radiation (<50-100 mGy) over prolonged periods of time that lead to cellular dysfunction [6]. These changes can be more difficult to appreciate until they have become a developing neoplastic process. Management of these changes is dependent on the type of lesion. Both processes can best be mitigated by using protective equipment such as lead aprons, a thyroid cover, protective gloves, leaded glasses, N95 or powered air-purifying respirators, wearing a dosimeter, and completely avoiding exposure to radiation [8].

**Table 2:** Stochastic versus deterministic effects of radiation exposure

Chang et al. (2021) [6]	Stochastic Effects	Deterministic Effects
Dosage	Low dose (<50-100 mGy)	High dose (>100 mGy)
Time	Long	Short
Threshold	Not present	Present
Probability	Increases with dose	1 for all cases
Severity	Independent of dose	Dose dependent
Consequences	Classically neoplastic processes	Hair loss, dermatitis, cataracts, acute radiation syndrome, etc.

The thyroid gland is the largest classical endocrine organ that has been extensively studied in relation to radiation exposure. However, there seem to be inconclusive findings when attempting to find a linear relationship between the two. Several studies have shown a dose-response relationship between low-dose (~0.7 Gy) radiation exposure and the development of autoimmune hypothyroidism [9,10]. However, these findings often fail to be replicated, leaving an inconsistent conclusion. However, moderate and high doses of radiation (0.37-55 Gy) have been shown to be unequivocally associated with autoimmune hypothyroidism and thyroid malignancies [8,11-13]. From this, it would be fair to conclude that, at certain dosages of radiation, the result is inflammatory damage to the thyroid stromal cells, which may encourage autoimmune and/or neoplastic development.

The prostate gland is another organ that has been studied with respect to radiation exposure and cancerous development. One study looked at low-dose radiation exposure from diagnostic X-ray procedures, which found a significant association between exposure to hip and pelvic X-ray and prostate cancer risk irrespective of family history, age, or social class [14]. A diagnostic X-ray will provide roughly 0.1 mSv (0.0001 Gy) of radiation. This shows the distinction of low-dose radiation, resulting in inflammation severe enough to drive neoplastic changes. This may be due to alterations in the prostate microbiome as well. Radiation may result in a reduction in the diversity of microorganisms that maintain the homeostatic environment of the prostate. When changed, there has been a relationship drawn between prostatitis and altered microbiomes. The result of chronic bacterial prostatitis, due to dysbiosis, may also increase the risk of prostate cancer [15].

The pituitary gland is an organ that has received less attention with respect to the development of inflammatory reactions and neoplastic processes in response to radiation exposure. While it is well documented that radiation exposure can result in malignancies of the brain, the pituitary is less associated with radiation-induced changes [7,16]. However, a study from the American Cancer Society in the 1950s showed that low-dose radiation (1.4 Gy) used to treat tinea capitis in children was associated with a 25% increase in the incidence of pituitary adenomas [17]. This continues to be an area of medicine that receives little attention. However, there are a few reasons that the pituitary has exceptional immunity from radiation-induced inflammation that could result in the malignant behavior of parenchymal cells.

Several studies have also looked at the relationship between radiation and the occurrence of soft tissue malignancies. Radiation-induced soft tissue changes are commonly reported in children exposed to low-dose radiation. Similar results have been documented because of primary malignancies subject to radiation treatment [17,18]. In addition, there may be an increase in the risk of tendon rupture due to the weakening of soft tissue with prolonged radiation exposure. The literature seems to be inconclusive about whether low-dose radiation has any detrimental effect on articular cartilage and other soft tissue ligaments and tendons [19,20]. However, with excess exposure to radiation, the potential for inflammatory damage would still be present [20]. The inflammatory damage should, therefore, come into question when discussing the severity of damage and whether the tendons and ligaments are subject to permanent detriment that could increase susceptibility to rupture.

After an investigation of the patient's past medical history and family history, there was a strong suspicion that these findings were a result of long-term radiation exposure. Investigation of primary genetic mutations is a crucial portion of this work-up. Many of these genetic syndromes including, but not limited to, Li Fraumeni and MEN could mimic neoplastic processes driven by radiation exposure. Therefore, ruling them out primarily is important in making a diagnosis of radiation-driven neoplasms [6]. The concurrence of both may also be evident. Primary tumor syndromes driven by tumor suppressor genes are subject to the “two-hit hypothesis” model, where both genes are required to mutate for a neoplastic process to take place. One is typically inherited, and the second mutation may be random or driven by outside stressors, such as radiation [21]. Therefore, radiation exposure, especially over a long period of time, would increase the odds of a "second hit" occurring.

After discussing the implications of chronic radiation exposure and its potential effects on various organs and tissues, strategies for monitoring and mitigating such risks are crucial to consider. One tool for monitoring radiation exposure is the Geiger counter, also known as a Geiger-Muller counter [22]. This portable device detects and measures ionizing radiation, providing valuable information about ambient radiation levels in the environment. In populations with potential exposure to radiation, regular monitoring with Geiger counters can help assess and manage the risk of radiation-related health effects. This monitoring can be done on a healthcare level as well as an individual level. These devices allow real-time monitoring of radiation levels, which can help prompt action to minimize exposure and possible consequences of low-dose radiation. Thus, implementing Geiger counters into safety protocols on an individual level may help reduce the end-organ damage seen with radiation exposure.

When discussing radiation-induced inflammatory changes to tissue, the prior examples were observed in this patient. Though some of them are commonly reported in literature, a few of them are not well represented. We bring attention to this, as the inflammatory changes seen in our case may be appropriate to begin compiling more data. Doing so would ultimately aid in quantifying and characterizing the impact that radiation exposure may have on these tissues.

## Conclusions

We present a case of a 66-year-old male who grew up in an area well known for heavy radiation exposure due to uranium mining. Given the prolonged exposure to low-dose radiation combined with his current presentation, the role that radiation exposure played is worth discussing. The constellation of pathologies as a result of chronic low-dose radiation must be taken into consideration. These diseases are not common to observe in one person absent of other severe chronic illnesses.

We present this case to bring awareness to the long-term effects of low-dose radiation exposure in developing children. There are many populations that live in or around previous uranium and radon mining demographics. As in this case, the laboratories and bustling towns continue to function. While mining and processing may have ceased, the effects linger on. The literature on the impact of such exposure is scarce. This would beg the question of the need to potentially begin screening for this etiology at younger ages so that preventative practices and management could be assumed earlier and long-term complications could be avoided.
